# Supplementary material for: SyNDI: synchronous network data integration framework
Source: BMC Bioinformatics. 2018 Nov 6;19:403. doi: 10.1186/s12859-018-2426-5 (PMC6219086; doi:10.1186/s12859-018-2426-5)
Supplement: Supplementary file 5 — Files of the example presented in Additional file 4. This additional file is a zip package that contains all files used in Additional file 4. Detailed descriptions of each of these files are presented in a README file included in this zip package. (GZ 30411 kb) [file 12859_2018_2426_MOESM5_ESM.gz › SyNDITools/Meme2Fimo/meme_4.9.0_4.tar.gz!/meme_4.9.0/doc/chen2meme.html]

chen2meme


# chen2meme

**Usage:** `chen2meme [options]`

**Description:**

Read a CHEN matrix file and convert to MEME format. You may concatenate
files for more than one motif.

**Input:**

Reads `standard input`.

Concatenated CHEN files describe one or more motifs. Each motif
has a header line and a position-weight matrix.

A probability matrix and optionally a log-odds matrix are output for each
motif. The probability matrix is computed using
pseudo-counts consisting of the background frequency (see -bg, below)
multiplied by the total pseudocounts (see -pseudo, below). The log-odds
matrix uses the background frequencies in the denominator and is log
base 2.

**Options:**

-skip <ID>
:   skip this ID (may be repeated)

-bg <background file>
:   file with background frequencies of letters; default: uniform background

-pseudo <total pseudocounts>
:   add <total pseudocounts> times letter background to each frequency; default: 0

-logodds
:   print log-odds matrix, too; default: print frequency matrix only

-url <website>
:   website for the motif; The motif name is substituted for MOTIF\_NAME;

-h
:   print usage message

**Output:**

Writes MEME format to standard output.

**Sample Input:**

```
>NANOG
2       64      4       10
1       66      1       12
52      1       0       27
0       0       0       80
0       0       0       80
0       5       74      1
3       1       1       75
3       49      14      14
57      9       0       14
1       0       3       76
1       3       46      30
4       48      12      16
30      16      3       31
31      12      19      18
41      12      12      15
16      20      12      32
>OCT4
11      216     13      99
168     10      1       160
1       6       2       330
12      0       8       319
21      92      213     13
98      5       5       231
68      93      37      141
326     0       4       9
0       0       0       339
0       0       319     20
4       289     19      27
288     1       1       49
212     25      73      29
294     8       23      14
55      28      54      202
```
